# MassBank: an open and FAIR mass spectral data resource

**DOI:** 10.1093/nar/gkaf1193

**Published:** 2025-11-11

**Authors:** Steffen Neumann, René Meier, Michael Wenk, Anjana Elapavalore, Takaaki Nishioka, Tobias Schulze, Michael Stravs, Hiroshi Tsugawa, Fumio Matsuda, Emma L Schymanski

**Affiliations:** Computational Plant Biochemistry, Leibniz Institute of Plant Biochemistry, Halle 06120, Germany; German Centre for Integrative Biodiversity Research (iDiv) Halle–Jena–Leipzig, Leipzig 04103, Germany; Computational Plant Biochemistry, Leibniz Institute of Plant Biochemistry, Halle 06120, Germany; Computational Plant Biochemistry, Leibniz Institute of Plant Biochemistry, Halle 06120, Germany; Luxembourg Centre for Systems Biomedicine, University of Luxembourg, 6 avenue du Swing, Belvaux 4367, Luxembourg; The Mass Spectral Data Working Group of the Mass Spectrometry Society of Japan. Yamabuki-cho 332-6, Shinjuku-ku, Tokyo 162-0801, Japan; Helmholtz Centre for Environmental Research—UFZ, Permoserstraße 15, Leipzig 04318, Germany; Eawag: Swiss Federal Institute of Aquatic Science and Technology, Dübendorf 8600, Switzerland; TOFWERK AG, Thun 3645, Switzerland; Department of Biotechnology and Life Science, Tokyo University of Agriculture and Technology, 2-24-16 Naka-cho, Koganei, Tokyo184-8588, Japan; Department of Bioinformatic Engineering, Graduate School of Information Science and Technology, The University of Osaka. 1–5, Yamadaoka, Suita, Osaka 565-0871, Japan; Luxembourg Centre for Systems Biomedicine, University of Luxembourg, 6 avenue du Swing, Belvaux 4367, Luxembourg

## Abstract

The open spectral library MassBank (https://massbank.jp/) started in 2006 in Japan, as one of the first open source and open access cross-vendor mass spectral libraries. The first dedicated European MassBank server (https://massbank.eu/) was launched in 2011, adding spectra from compounds of environmental relevance to complement the original metabolomics focus. Recent developments boosted the FAIRness of MassBank data, and a redesigned web interface with modern architecture was launched in 2025. All records are under version control, and versioned releases are pushed to an independent data repository with digital object identifier assigned to each data release. Semantic metadata integrates the spectral data into the world of linked open data. The record format enables many downstream studies, and records are validated by an automated pipeline to ensure data quality upon submission and review existing records. The SPectraL hASH was added for spectral content-based access. MassBank is cross-integrated in several resources such as MassBank of North America, Global Natural Product Social Molecular Networking, PubChem, the US EPA CompTox Dashboard, NORMAN Database System, RforMassSpectrometry, and more. MassBank now boasts a total of 119 845 spectra of 18 529 compounds from 53 contributors from around the world.

## Graphical Abstract

**Graphical Abstract:**
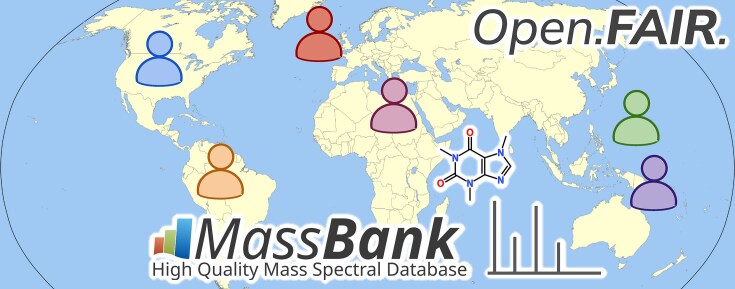

## Introduction

Mass spectrometry (MS) is the analytical method of choice to analyze complex samples due to unmatched sensitivity and selectivity in biological and environmental matrices. It is used in many scientific domains, including metabolomics, exposomics, marine, biomedical, biotechnology, and environmental research. Depending on the application and available resources, different types of low- and high-resolution MS instrumentation, often coupled to chromatographic separation, are used. In the context of this article, the main purpose of MS is the identification of analytes (e.g. small molecules such as metabolites, lipids, pharmaceuticals, pesticides, or persistent organic pollutants) supported by mass spectral library search. Although “small molecules” are defined differently in various domains, for practical purposes in this article, this refers to molecules below ∼2000 Da.

Several general and domain-specific low- and high-resolution mass spectral (HRMS) libraries are available as commercial, open access, or in-house libraries. Notable collections include the 2023 NIST MS (394k spectra of 347k compounds) and Tandem MS libraries (2.4M spectra of 51k compounds) [1], METLIN with HRMS spectra of >931k compounds [2], and the spectral libraries in the GNPS ecosystem [3]. The coverage of commercial and open access libraries, including both NIST and METLIN, was explored in 2016 [4], but has been superseded with recent content expansion, especially of METLIN.

MassBank is an open source and open access mass spectral library with a strong focus on the metabolomics and environmental chemistry domains. It was founded in 2006 at the website https://massbank.jp/ by the JST/BIRD project in Japan and then supported by the Mass Spectrometry Society of Japan (MSSJ) [5]. In 2008, the Leibniz Institute of Plant Biochemistry (IPB Halle) joined the MassBank consortium as the first European site, followed by the NORMAN Association in 2011. The European server https://massbank.eu/ is currently hosted at the Helmholtz Centre for Environmental Research (UFZ) in Germany. In 2016, the main software development moved to the IPB Halle, with the Luxembourg Centre for Systems Biomedicine (LCSB) maintaining the links to NORMAN and cross-resource integration. Although MassBank of North America (MoNA) contains the MassBank name, this is an independent resource hosted by the Fiehn Lab in the USA (https://mona.fiehnlab.ucdavis.edu/) and is not the focus of this article. The Japanese and European servers, in contrast, mirror the same user-contributed content. Users can access the full range of available MassBank records on the websites of MassBank Europe at https://massbank.eu/ and MassBank Japan at https://massbank.jp/. It is also possible to self-host an institutional instance if the data can not be made publicly available, e.g. in cases requiring intellectual property protection. All code and spectral records are available on the MassBank Consortium GitHub page (https://github.com/MassBank/). This update article describes the updates to the MassBank ecosystem and spectral content over the last two decades.

## Materials and methods

All the spectral data collected in MassBank are submitted by the users, not copied from other mass spectral databases. MassBank has two policies for collecting mass spectral data. First, MassBank accepts new mass spectra of a chemical compound even when mass spectra of the same chemical compound are already deposited. Second, MassBank recommends its contributors to deposit mass spectra that analyze the same chemical compound using different collision energies and ionization modes, where feasible. Since MassBank includes mass spectra where the precursor ion is not observed directly in the spectrum, precursor information is also included in a dedicated field in the record information. These principles enhance the reliability and utility of the MassBank data.

Records are stored as text files under version control in the MassBank-data repository, following the record format specifications. The MassBank record format (current version 2.6.0) is defined in the documentation and includes mandatory and optional fields together with examples. All changes to the record format are documented, including the rationale for the change. New fields are discussed within the consortium and contributors via GitHub issues. The flexibility and richness of the MassBank record format allow a wide range of contributions to MassBank, effectively enabling any mass spectrum to be shared. A validation system is in place to check new records and retrospectively screen existing records for compatibility with the record format and consistency of chemical information for individual records. The validation can also process the entire MassBank in batch mode.

Structural information associated with the mass spectral record is directly included in the record. Unlike earlier MassBank versions, separate mol files are no longer needed during submission, and information is taken directly from the SMILES field of the record files. This has enabled the inclusion of spectra from tentatively or partially identified compounds, such as metabolites or transformation products that are detected in experiments and may be positional isomers, yet were not available in sufficient amounts for isolation and complete confirmation [6]. In these cases, records are marked as “tentative,” and the structure can be expressed as SMARTS, extended SMILES or CxSMILES, which can be rendered with CDK Depict. An initiative to develop International Chemical Identifiers (InChIs) for such cases has been proposed but not yet implemented [7]. Since the 2025.05.1 release, records are also enhanced with ChemOnt (ClassyFire) classifications [8], which can be used when browsing or searching MassBank.

The annotation of mass spectral peaks is achieved through the PK$ANNOTATION field, which appears separately to the peak list. Records prepared using RMassBank [9] are annotated with a proposed peak formula, the number of possible formulas, a mass error and ppm error. Since structural information associated with each fragment can provide richer user information for identification purposes, some contributors such as MSSJ have provided structure annotations calculated using MS-FINDER [10] since 2022 (see e.g. MSBNK-MSSJ-MSJ04174).

MassBank is open source and open access, with all related code gathered under the MassBank Consortium on GitHub (https://github.com/MassBank/). This includes all data, with records under each contributor (https://github.com/MassBank/MassBank-data), documentation (https://github.com/MassBank/MassBank-documentation), the current re-designed user interface, and backend architecture (https://github.com/MassBank/MassBank3), processing software such as RMassBank [9, 11] (RRID:SCR_002797) and more. The MassBank ecosystem builds on many other open source projects, including the Chemistry Development Kit (CDK) [12], matchms [13], Bingo [14], and OpenChemLib JS [15].

## Results

**Figure 1.**
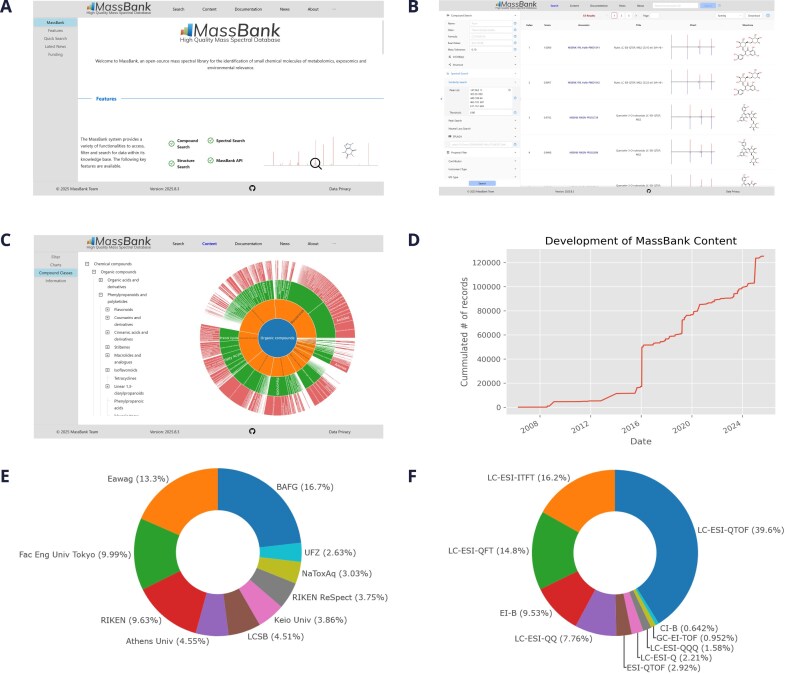
(**A**) The MassBank web frontend: start page with quick access, (**B**) example spectral search results, (**C**) browsing by compound classes, (**D**) growth of MassBank content over the last 20 years, (**E**) the top 10 contributing institutions, and (**F**) the top 10 instruments. Panels (A–C, E, and F) are screenshots of MassBank web pages, and panel (D) was created using the Jupyter Notebook in MassBank-analytics.

MassBank (release 2025.05.1) currently contains 119 845 unique spectra by SPectraL hASH (SPLASH) [16] of 18 529 unique compounds by InChI [17] from 53 contributors. The current view is shown in Fig. 1 A and B, and the ChemOnt overview in Fig. 1C. MassBank continues to grow with new contributions and contributors, as shown in Fig. 1D.

An overview of the statistics is given in Fig. 1D–F and on the content pages. The top 10 contributors include BAFG (the large spike in spectra in 2024; see Fig. 1D and E), Eawag (several contributions, including a large one in 2016; clearly visible in Fig. 1D), University of Tokyo and RIKEN (both original contributors), Athens University, LCSB, Keio University, RIKEN ReSpect, NaToxAq project, and UFZ. The metabolomics and environmental contributions, as well as distribution of European and Japanese contributions are still reasonably well balanced. Despite the European and Japanese dominance in the top 10, the global reach of MassBank has grown and includes contributions from Canada, the United States of America, China, Brazil, and Peru.

The Mass Spectral Data Working Group of the MSSJ has been collecting mass spectra from researchers for the past decade. The group has compiled 2872 MassBank records with a broad variety of compound classes including plant secondary metabolites (e.g. MSBNK-MSSJ-MSJ00001) and environmental pollutants (e.g. MSBNK-MSSJ-MSJ04182). To encourage data submission, the working group has collaborated with the journal “Mass Spectrometry (Tokyo)” to publish papers detailing the submitted spectral data [18]. Furthermore, import and export functions for the MassBank record format have been integrated into the data processing software MS-FINDER [10], facilitating the creation of these records.

In terms of spectral data, MassBank is 82.3% tandem MS (MS2) data, with 16.8% MS data (primarily electron impact spectra, rather than MS1 data associated with MS2 spectra) and small amounts of MS3, MS4 and, MS^*n*^ spectra (0.75%, 0.06%, and 0.02%, respectively). Most spectra (71.2%) are positive mode, with 28.8% of spectra in negative mode. The dominating instrument types (see Fig. 1F) are high-resolution quadrupole time of flight (QTOF) machines coupled to liquid chromatography (LC) with electrospray ionisation (ESI) (LC–ESI–QTOF, 39.6%) followed by LC–ESI Orbitraps (LC–ESI–ITFT, 16.2% and LC–ESI–QFT, 14.8%), magnetic sector electron impact instruments (EI-B, 9.5% and LC–ESI–QQ, 7.8%). Nevertheless, MassBank also includes several exclusive collections of alternative ionisation spectra, including matrix-assisted laser desorption/ionization, atmospheric pressure chemical ionisation, and chemical ionisation spectra (full listing under the “instrument type” tag in the content pages). There are discussions to expand this further to include pyrolysis GC spectra, which will require additional adjustment to the record format for this special case.

While most of MassBank is spectra recorded on reference standards or isolated compounds, MassBank also accepts spectra from complex samples. The COMMENT: CONFIDENCE tag, first used in RMassBank-contributed records, was formally introduced into the record format in 2018 to capture these cases. Of the 65 538 spectra with this tag, 64 349 (98.2%) are reported as being from standard references, isolated or synthesized compounds (32 372 spectra), or as confidence level 1 according to the metabolomics standard initiative [19] or Schymanski *et al.* [20] schemes (31 977 spectra). Only 251, 644, and 31 records claim lower identification levels 2, 3, or 4, respectively, while another 238 spectra are recorded of being of biological origin. This total of 1164 records currently comprises <0.02% of all records with this tag.

MassBank-hosted spectra are also available on other open mass spectral libraries, including GNPS [3] and MoNA. They are also integrated in PubChem [21, 22], CompTox Chemical Dashboard [23], the NORMAN Database System [24] including the NORMAN Suspect List Exchange (NORMAN-SLE) [25], the NFDI4Chem search service, which indexes databases and repositories in the NFDI4Chem data federation, and WikiData [26]. Through the cross-population in other open mass spectral libraries, MassBank spectra are now accessible within several metabolomics and exposomics software systems, including MS-DIAL [27, 28], MZmine [29, 30], patRoon [31, 32], MetFrag [33], and RforMassSpectrometry [34]. Some vendors also offer support for MassBank: for instance the Thermo Fisher Scientific library manager mzVault enables the direct import of MassBank records for reuse, while Agilent offers an export function for users to contribute to MassBank. The MassBank data releases include exports of all spectra in JSON and two flavours of the popular MSP format, for better downstream integration.

## Discussion

The FAIR principles [35] promote data to be findable, accessible, interoperable, and reusable. MassBank has adapted the software ecosystem and record format to address these principles to a very high standard: spectra are findable via the rich chemical and analytical metadata, and addressable directly on the MassBank web sites, but also through the identifiers.org and bioregistry.io resolvers via the standard HTTP(S) protocol. The metadata for all records in each data release is available in the SchemaOrg format, both directly from the MassBank website and machine readable REST interface and also from GitHub and Zenodo for long-term accessibility (see the “Data availability” section). All records include explicit license information. The rich requirements of the format create a higher initial contribution barrier, especially compared with other resources such as GNPS and MoNA, but enable downstream studies not possible as easily with other libraries [21, 36–38].

MassBank has become a key resource in the highly connected ecosystem of life science infrastructures. The first 10 years of development were primarily driven by the Japanese members. The early European efforts were supported by the NORMAN Association as part of the NORMAN database system and through the contributions of research groups at the IPB Halle, UFZ, Swiss Federal Institute of Aquatic Science and Technology (Eawag), and LCSB, see the “Funding” section. The NORMAN Association has been instrumental in bringing in several of the very large contributions in recent years through environmental networks, helping expand the image beyond that of a metabolomics database. This investment has paid off with the growing interest in exposomics in biomedical research, such that MassBank now also serves as a valuable open resource for spectra of environmental relevance.

Since 2018, MassBank development has been supported by the German Network of Bioinformatics Infrastructures (de.NBI), which is the German node of the European ELIXIR life science infrastructure. This allows the inclusion in ELIXIR resources such as identifiers.org and FAIRsharing. MassBank is currently being nominated as an ELIXIR Community Database, which promises even wider adoption and more community contributions. MassBank is the official database of MSSJ. Additionally, the Shin(Neo)–MassBank Project is currently underway in Japan to enrich MassBank data (https://shin.massbank.jp/).

## Conclusions

MassBank (https://massbank.jp/ and https://massbank.eu/) proved sustainable operation and thrived scientifically now for two decades. In that time, it has become a key resource for the open exchange of spectral data, feeding into other databases, resources, and software relevant for small molecule identification efforts. Contributions and contributors are growing, and in addition to providing spectra of natural products and metabolites for the life sciences, MassBank is becoming an important source of environmental compounds of relevance, as researchers are showing increasing willingness to exchange data to improve open workflows. New contributions are very welcome (see https://massbank.eu/MassBank/documentation).

## Data Availability

All records in MassBank.EU and MassBank.JP are managed and released through GitHub (https://github.com/MassBank/MassBank-data), Zenodo, and the SoftwareHeritage Archive, e.g. under swh:1:dir:6385d240fc0d8840dc22ec7d114c31aef685ea8d. Each MassBank release is tagged with a version number and digital object identifier (DOI) to enhance reproducibility, e.g. release version 2025.05.1 is packaged as MassBank-data/releases/tag/2025.05.1, archived on Zenodo [39], and on SoftwareHeritage as swh:1:dir:6385d240fc0d8840dc22ec7d114c31aef685ea8d. The entire MassBank software stack is available under an open source license from https://github.com/MassBank. The MassBank3 [40], MassBank-similarity-api [41], and MassBank-export-api [42] repositories are needed to compile and install a local MassBank system. All software are also built as Docker containers and available from https://quay.io/organization/massbank and can be launched via docker-compose or installed into a Kubernetes cluster with Helm charts from MassBank-charts [43].

## Notes

Present Address: German Environment Agency, II 2.5—Laboratory for Water Analytics, Colditzstraße 32, 12099 Berlin, Germany
